# Normal Aging Modulates the Neurotoxicity of Mutant Huntingtin

**DOI:** 10.1371/journal.pone.0004637

**Published:** 2009-02-27

**Authors:** Elsa Diguet, Fanny Petit, Carole Escartin, Karine Cambon, Nicolas Bizat, Noëlle Dufour, Philippe Hantraye, Nicole Déglon, Emmanuel Brouillet

**Affiliations:** 1 Commissariat à l'Energie Atomique (CEA), Institut d'Imagerie Biomédicale (I2BM), Molecular Imaging Research Center (MIRCen), Orsay, France; 2 Centre National de la Recherche Scientifique (CNRS), Unité de Recherche Associée CEA-CNRS 2210, Orsay, France; Swiss Federal Institute of Technology Lausanne, Switzerland

## Abstract

Aging likely plays a role in neurodegenerative disorders. In Huntington's disease (HD), a disorder caused by an abnormal expansion of a polyglutamine tract in the protein huntingtin (Htt), the role of aging is unclear. For a given tract length, the probability of disease onset increases with age. There are mainly two hypotheses that could explain adult onset in HD: Either mutant Htt progressively produces cumulative defects over time or “normal” aging renders neurons more vulnerable to mutant Htt toxicity. In the present study, we directly explored whether aging affected the toxicity of mutant Htt *in vivo*. We studied the impact of aging on the effects produced by overexpression of an N-terminal fragment of mutant Htt, of wild-type Htt or of a β-Galactosidase (β-Gal) reporter gene in the rat striatum. Stereotaxic injections of lentiviral vectors were performed simultaneously in young (3 week) and old (15 month) rats. Histological evaluation at different time points after infection demonstrated that the expression of mutant Htt led to pathological changes that were more severe in old rats, including an increase in the number of small Htt-containing aggregates in the neuropil, a greater loss of DARPP-32 immunoreactivity and striatal neurons as assessed by unbiased stereological counts.

The present results support the hypothesis that “normal” aging is involved in HD pathogenesis, and suggest that age-related cellular defects might constitute potential therapeutic targets for HD.

## Introduction

Huntington's disease (HD) is an autosomaly inherited neurodegenerative disorder characterized by abnormal involuntary movements (chorea), cognitive decline and psychiatric symptoms associated with neurodegeneration, which affects mainly the striatum [1]. HD is caused by an expansion of CAG trinucleotide repeats in the *HD* gene that codes for huntingtin (Htt) [2]. When glutamine (polyQ) repeats located in the N-terminal part of Htt are higher than approximately 38 repeats, they cause disease, while unaffected individuals can have repeats lengths of up to 35 repeats. The age of onset is inversely correlated with the number of CAG/polyQ repeats [3], with, however, considerable inter-individual variation within a given repeat-length range, suggesting the importance of other genetic and environmental factors [4].

Although “normal” aging is thought to play a role in neurodegenerative diseases (e.g. Parkinson's and Alzheimer's disease) [5], the experimental evidence for this phenomenon in HD is sparse. In HD patients, the probability of disease onset increases with age, indicating that aging is a risk factor. However, the mechanisms by which aging might play a role in HD are speculative. “Normal” aging may render striatal neurons more vulnerable to mutant Htt toxicity. In support of this hypothesis, cellular functions known to be altered by aging, including the anomalies of the ubiquitin/proteasome pathway [6], Ca^2+^ deregulation [7], and oxidative stress [8], are likely to be involved in HD pathogenesis [9]. Alternatively, the adult onset of HD may result from the progressive accumulation of toxic Htt fragments. In this case, cellular defects related to “normal” aging would not “accelerate” degeneration in HD. In support of this view, one study has suggested that the probability of degeneration may be constant in HD [10].

The lack of direct evidence that aging-related processes could play a role in HD is mainly a result of an absence of experimental settings *in vivo* where the influence of aging on the toxicity of mutant Htt can be directly assessed. In most transgenic models of HD, mutant Htt expression takes place throughout life, and it is very difficult to dissociate the effects of “normal” aging by themselves from an age-related accumulation of defects specifically due to the presence of mutant Htt. The aging issue in HD is of importance since, if cellular defects related to “normal” aging are risk factors for HD, they could constitute interesting therapeutic targets to delay disease onset and slow progression.

In the present study, we used a rat model of HD [11]–[15] in which striatal degeneration is induced by lentiviral vectors encoding the 171 N-terminal amino acids of human Htt with an 82 CAG-repeat pathological expansion (Htt-171-82Q), in order to directly compare the neurotoxicity of mutant Htt in young and old animals. Our results show that the vulnerability of striatal neurons to mutant Htt increases with age.

## Results

### Aging does not modify PGK promoter efficacy

**Figure 1:**
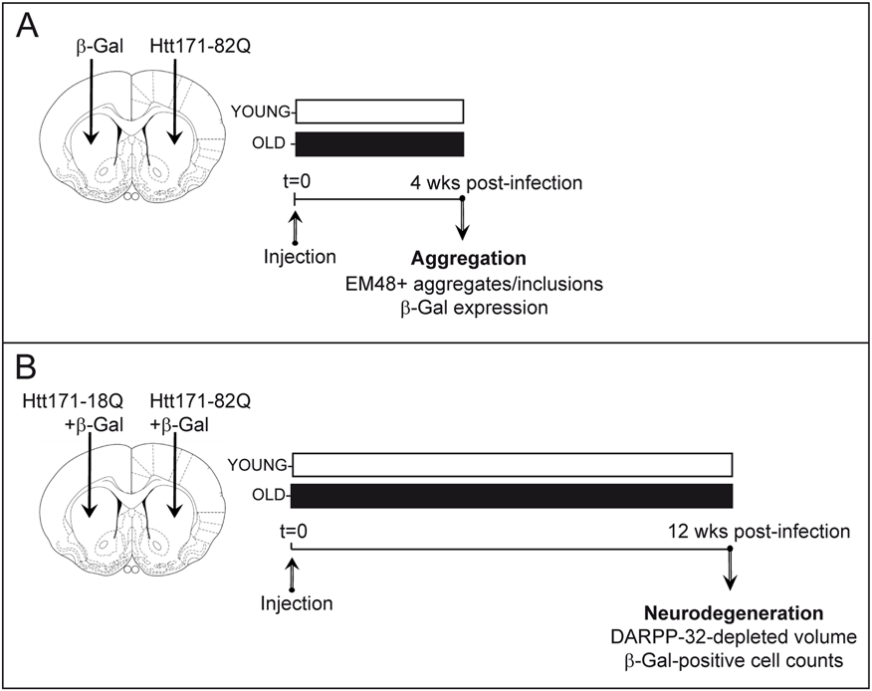
Experimental design to study age-dependent vulnerability of the striatum to mutant huntingtin (Htt). Young (3 week old) and old (15 month old) rats were injected with a lentiviral vector encoding the 171 N-terminal amino acids of mutant huntingtin with 82 polyglutamine repeats (Htt171-82Q), the corresponding wild-type fragment with 18 polyglutamine repeats (Htt171-18Q) or the reporter protein β-Galactosidase (β-Gal). In the first experiment (*A*), young and old rats received a stereotaxic injection of lentiviral vectors (2 µl, 200 ng/µl of p24) encoding β-Gal (left striatum) or Htt-171-82Q (right striatum). Histological evaluation was carried out 4 weeks after infection to determine the effects of aging on the expression of β-Gal and Htt171-82Q. In a second experiment (*B*), the actual degeneration produced by Htt171-82Q was characterized at a later time point after infection. Animals were injected with lentiviral vectors (4 µl, 200 ng/µl of p24) encoding β-Gal mixed with lentiviral vectors encoding either Htt171-82Q or Htt171-18Q. Histological characterization of the striatum consisting of an assessment of DARPP-32, EM48, and β-Gal immunoreactivity and activity was carried out 12 weeks post-infection. Unbiased stereological count of β-Gal-positive neurons were used to compare Htt-171-82Q toxicity with that of Htt171-18Q. Analysis of β-Gal levels in neurons in the Htt171-18Q-expressing striatum permitted the verification of the effect of age on transgene promoter (PGK) efficiency.

**Figure 2:**
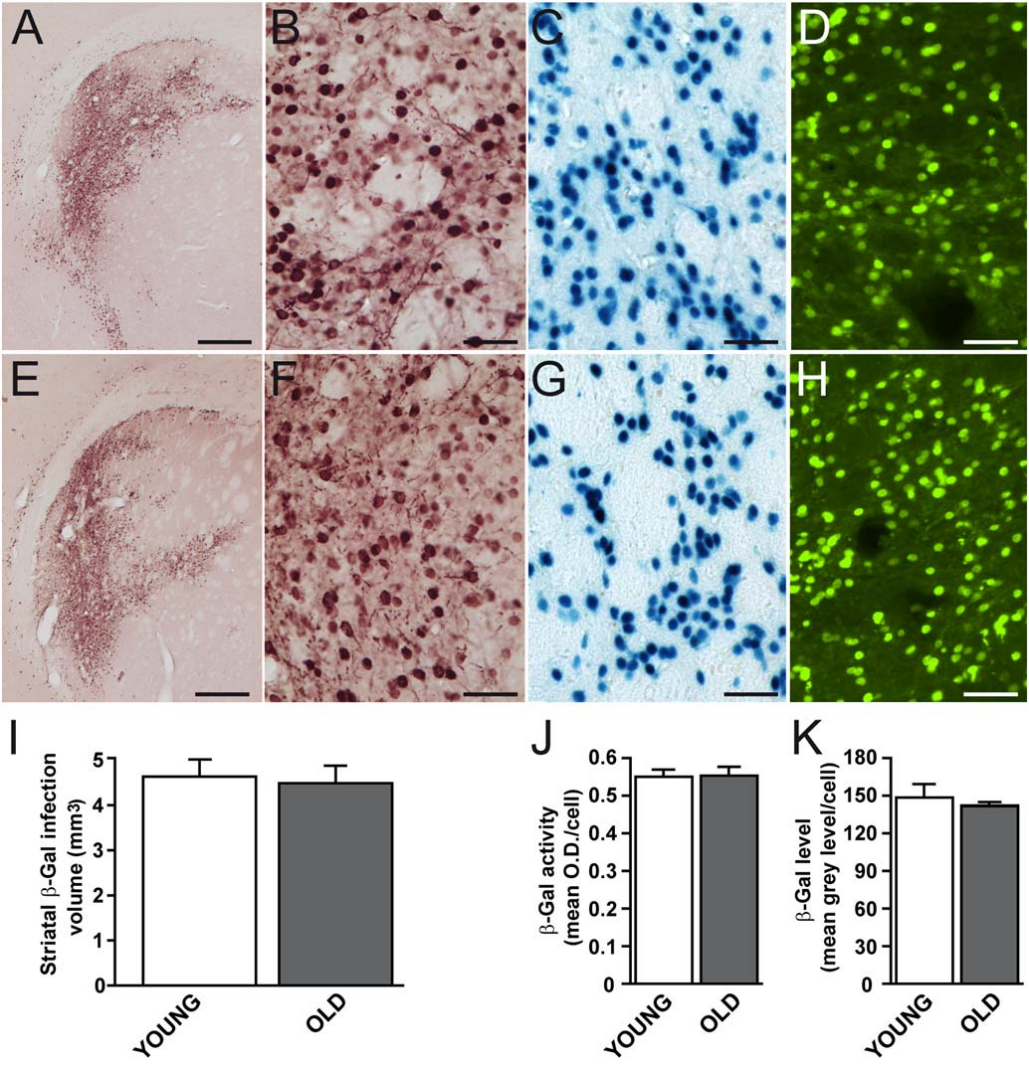
Absence of an effect of age on PGK promoter function. Young (3 week) and old (15 month) rats received stereotaxic injections of lentiviral vectors encoding β-Gal under the PGK promoter into the striatum. Histological evaluation was carried out 12 weeks after infection to determine the levels of expression of β-Gal using immunohistochemistry (*A, B, E, F*), β-Gal activity (*C, G*) and immunofluorescence (D, H). Typical photomicrographs showing the striatum after infection with lentiviral vectors encoding β-Gal in young (*A–D*) and old (*E–H*) rats. No major qualitative difference could be detected between young and old rats. *I*, quantification of the volume of the striatum expressing β-Gal. *J*, Quantification of β-Gal activity. *K*, Quantification of immunoreactivity using fluorescence detection. Note the absence of an effect of age. Scale bar = 500 µm for *A, C* and 50 µm for *B–D* and *F–H*. Results are expressed as mean+/−SEM. These are no significant differences as assessed by Student *t* test.

We wanted to determine the effect of aging on mutant Htt toxicity using lentiviral vectors (Fig. 1). We first examined whether aging could modify the transduction efficiency of our lentiviral vectors and/or change the efficacy of the promoter driving the expression of mutant Htt. As controls, lentiviral vectors encoding the reporter protein β-Galactosidase (β-Gal), which was modified to contain a nuclear localization signal (nls), were stereotactically injected alone (first experiment) or with wild-type Htt (second experiment). These experiments were designed to evaluate the efficacy of the PGK promoter in young (3 week) and old (15 month) rats (Fig. 1). β-Gal was detected in situ using immunohistochemistry, and immunofluorescence (Fig. 2). β-Gal enzymatic activity was also used to detect transduced cells and quantify the level of protein expression (Fig. 2). Qualitatively, there was no obvious age-related difference in the appearance of neurons expressing β-Gal (Fig. 2). Nuclei were densely stained. Cell bodies and processes, mostly dendrites with spines, were more rarely visible. At 12 weeks post-infection (i.e. when young rats were 12 weeks old and older rats were 18 months old), the volume of the striatum containing β-Gal-positive cells was not statistically different between the two age groups. Similar experiment was performed at 4 weeks post infection, showing no differences between old and young rats. Enzymatic detection of β-Gal activity showed similar labeling in striatal cells in young and old rats (Fig. 2C, G, J). Consistent with this, analysis of the levels of expression in transduced cells using immunofluorescence detection of β-Gal showed no difference between the two groups (Fig. 2D, H, K). This demonstrates that neither the transduction properties of lentiviral vectors nor the apparent efficacy of the PGK promoter used to express β-Gal, Htt171-82Q and Htt171-18Q in the experiments described hereafter significantly changes with age.

### Characterization of EM48-positive inclusions/aggregates

**Figure 3:**
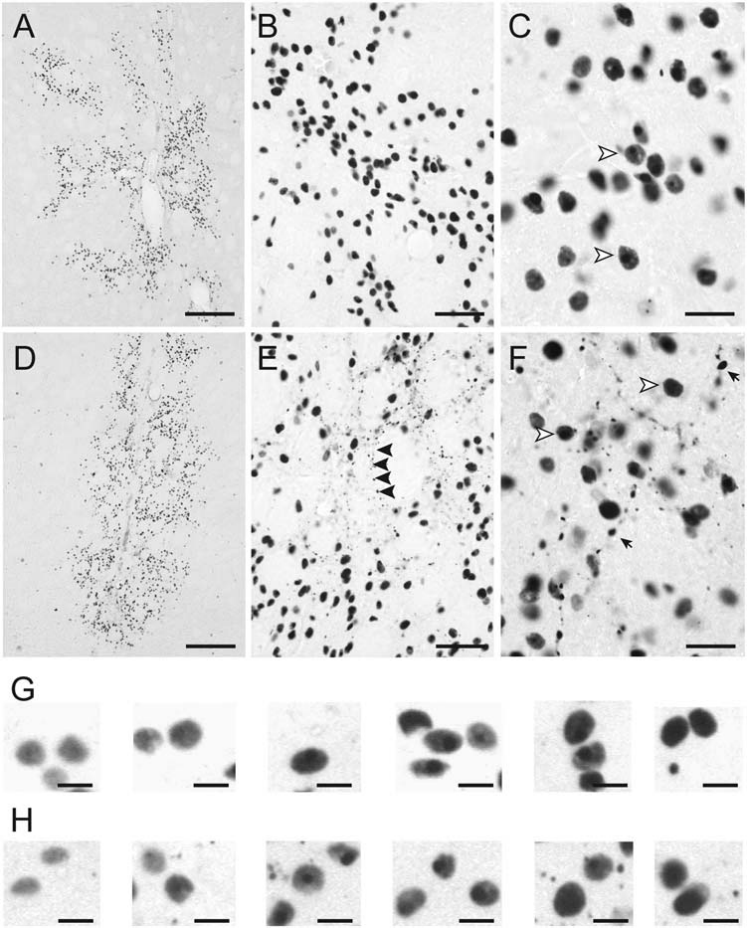
EM48-positive inclusions/aggregates in the striatum of young and old rats appear different. Young (3 week; *A, B, C*) and old (15 month; *D, E, F*) rats received a stereotaxic injection of lentiviral vectors encoding Htt171-82Q (2 µl, 200 ng/µl of p24) and β-Gal with the PGK promoter into the striatum. Histological evaluation was carried out 4 weeks after infection using EM48 immunohistochemistry to detect mutant Htt-containing aggregates and inclusions. Qualitatively, the striatal region showing EM48-positive inclusions/aggregates was similar in young (*A*) and old (*D*) rats (see table 1 for quantification). Higher magnification observation indicated that the nature of the aggregates was different between the groups (*B, C, G* and *E, F, H*). While nuclear inclusions (arrowhead) were essentially similar in both groups, many small aggregates (arrow in *E* and *F*) often organized as if located in processes are visible in old rats but not in young animals. *G, H*, examples of nuclear EM48 immunoreactivity displaying from a pale (left images) to dark (right images) diffuse staining, on which an inclusion is superimposed. Scale bar = 250 µm for *A, D*, 50 µm for *B, E*, 20 µm for *C, F*, and 10 µm for *G, H*.

Huntingtin-containing inclusions and aggregates were detected by immunohistochemistry using the EM48 antibody [16], [17]. As expected, EM48 immunoreactivity was absent in striata injected with the wild-type human Htt fragment (Htt171-18Q) in both age groups. In Htt171-82Q-injected striata, EM48 immunoreactivity was found at 4 weeks post-infection in both young and old rats. Typically, immunoreactive nuclei (apparent cross-sectional area of ∼50 µm^2^) showed a diffuse staining which was variable depending on the cells considered, appearing relatively pale in certain cases (left images, Fig. 3G, 3H) or quite dark in others (right images, Fig. 3C, F–H). Superimposed on this diffuse staining, a round strongly stained inclusion was generally seen that, in some cases, could be prominent (Fig. 3C–H). These typical features (called intranuclear inclusions hereafter) are highly consistent with the intranuclear inclusions previously described in the striatum of HD patients and transgenic HD mouse models including knock-in models, using the same antibody [16], [18], [19]. In young rats, EM48-positive (EM48+) objects mainly consisted of intranuclear inclusions. In old rats, in addition to the nuclear inclusions, numerous densely stained small EM48+ objects that were not superimposed on any background were also observed. These small aggregates with a cross-sectional area of ∼1–10 µm^2^ were found to be widespread throughout the neuropil, sometimes organized as if localized in neuronal processes (Fig. 3). These objects resembled neuropil aggregates described previously [16], [17]. Although it cannot be ruled out that a small proportion of these small EM48+ aggregates lacking a nuclear background were actually present in the nucleus, we believe that the majority were localized in the somatodendritic compartment. They are thus called neuropil aggregates hereafter. In young animals, these objects were very rarely seen.

Quantification of the EM48+ objects revealed no age-related differences in the volume of the striatum exhibiting EM48+ objects (Table 1). Similarly, a determination of the total number of EM48+ objects (inclusions/aggregates) using automated image acquisition and analysis systems indicated that neither the mean apparent numerical density nor the absolute number of Htt-containing aggregates within the striatum were markedly different between the two age groups. However, we observed that the mean cross-sectional area of the EM48+ objects (as determined by analyzing more than 10,000 objects per animal) was slightly (∼10%) but consistently and significantly reduced in older rats when compared with young animals (p<0.0001, Table 1).

**Table 1:** Quantitative characteristics of EM48-positive objects in young and old rats.

	Young rats	Old rats	
Striatal volume with EM48-positive objects (mm^3^)	2.88+/−0.48	3.06+/−0.56	n.s.
Striatal density of EM48-positive objects (counts/mm^2^)	1010.83+/−67.90	1100.33+/−58.30	n.s.
Number of EM48-positive objects (estimated total number)	71,640+/−11,268	80,905+/−12,244	n.s.
Cross-sectional area of EM48-positive objects (µm^2^)	56.34+/−1.08	47.74+/−0.94	p<0.0001

Rats received intrastriatal injection of lentiviral vectors encoding the Htt171-82Q at different ages (young, 3 weeks; old, 15 months). Histological evaluation using EM48 immunohistochemistry for inclusions and neuropil aggregate detection was performed at 4 weeks post-infection. EM48-positive objects (inclusions and aggregates) were counted using an automated acquisition and image analysis system. Note that in older rats there is a reduction in the cross-sectional area of EM48-positive objects and a trend towards an increased number of objects.

**Figure 4:**
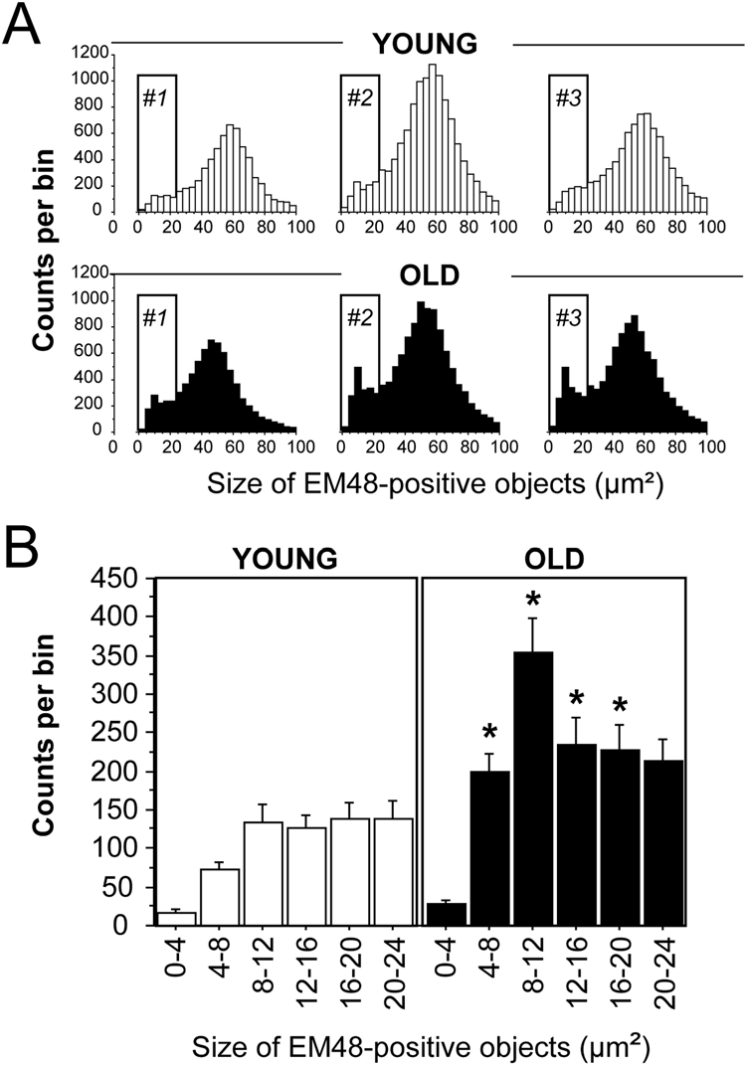
Morphometric analysis of EM48-positive inclusions/aggregates shows age-dependent differences. Young (3 week) and old (15 month) rats received a stereotaxic injection of lentiviral vectors encoding Htt171-82Q (2 µl, 200 ng/µl of p24) into the striatum. Histological evaluation was carried out 4 weeks after infection using EM48 immunohistochemistry and image analysis software. The number of EM48 positive objects was computed for 25 bins (steps of 4 µm^2^) based on cross-sectional area. *A*, typical individual distribution of object size is shown for 3 young rats (#1 to #3) and 3 old rats (#1 to #3). Note that the proportion of small aggregates (grey vertical bar) is higher in old animals than in young rats. *B*, the averaged distribution of small aggregates is shown for the two experimental groups (n = 6 per group). Note the clear cut augmentation of the number of aggregates with an apparent cross-sectional area in the 4–20 µm^2^ range in older rats. Note also that the distribution peak of EM48 positive objects in the 40–60 µm^2^ range was shifted to the left in older rats (∼46 µm^2^) as compared to young animals (∼58 µm^2^). A similar shift was found in all animals, consistent with a decreased nuclear inclusion size. Results are expressed as mean+/−SEM. *p<0.05, ANOVA and Bonferroni's *post hoc* test.

**Figure 5:**
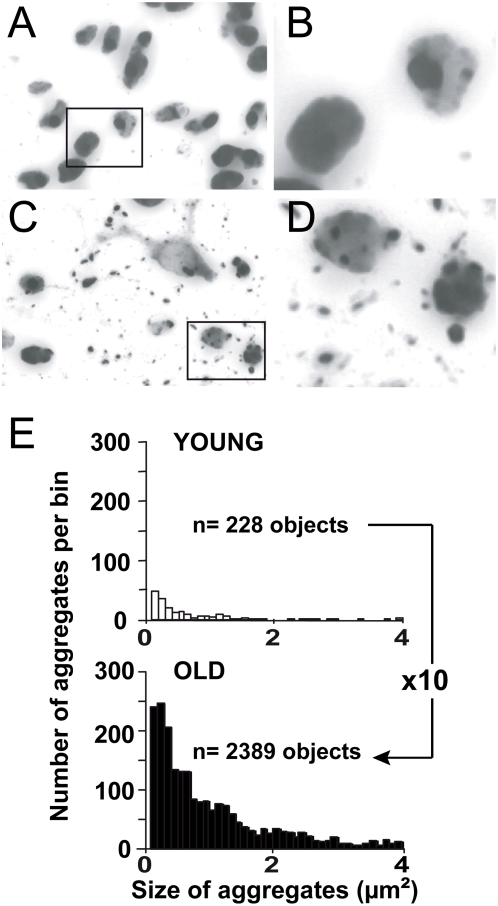
High resolution image analysis of EM48-positive aggregates indicates major age-dependent differences. Young (3 week) and old (15 month) rats received a stereotaxic injection of lentiviral vectors encoding Htt171-82Q (2 µl, 200 ng/µl of p24). Histological evaluation was carried out 4 weeks after infection using EM48 immunohistochemistry and image analysis software. The number of EM48 positive objects (aggregates) was determined from images obtained using a 50× objective at different focal depths (see Materials and Methods) so that very small EM48 positive objects could be detected. Typical images acquired at 50× objective in a young rat (*A*) and an old rat (*C*). *B* and *D* correspond to zoomed images of the rectangles in A and B. Note the high resolution of the images allows reliable detection of even small aggregates. *E*, Histograms showing the distribution of small objects as a function of size (apparent cross-sectional area) in two animals, indicating that the proportion of very small aggregates in old rats is approximately ten fold higher than in young animals.

We further explored possible morphological differences in the EM48+ inclusions/aggregates using object size distribution analysis. A comparison of the morphometric characteristics of the objects detected with a visual determination of their localization suggested that, regardless of the age group considered, large EM48+ objects (40–80 µm^2^) mostly corresponded to nuclear-like inclusions. Distribution histograms revealed that the peak in the distribution of the objects occurred around 54 µm^2^ in young rats. In older rats this peak was shifted towards smaller objects (i.e. ∼46 µm^2^) (Fig. 4). Frequency distribution of EM48+ objects according to size also clearly revealed that the number of small inclusions (4–24 µm^2^) was increased by approximately 3 times in all old rats, compared with the young ones (Fig. 4). Since this contrast was likely to have been underestimated at the magnification used (20× objective), we performed an analysis at a higher resolution [12]. Results showed that there were approximately 10 times more very small aggregates (0.2–4 µm^2^) in adult animals than in young animals (Fig. 5).

Thus, at 4 weeks post-infection, a time point at which mutant-Htt-induced cell loss is minimal in this model [11], we observed a clear cut increase in the number of small neuropil-like aggregates in older rats as compared to younger rats. This indicates age-related changes in mutant Htt processing/elimination, aggregation and/or transport.

### Loss of striatal DARPP-32 expression is greater in old rats

**Figure 6:**
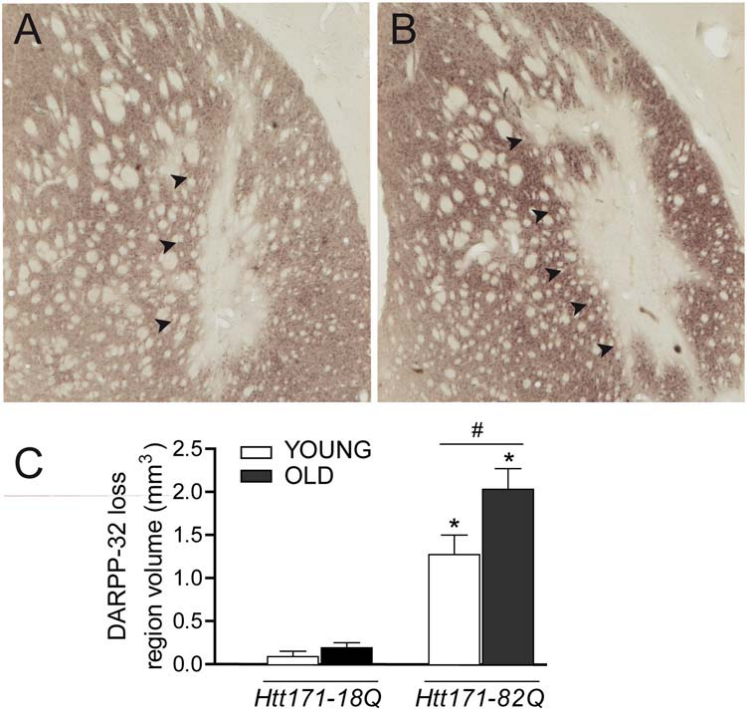
Loss of DARPP-32 induced by Htt171-82Q is more severe in old rats. Young (3 week) and old (15 month) rats received a stereotaxic injection of a mixture of lentiviral vectors (4 µl, 200 ng/µl of p24) encoding either Htt171-82Q and β-Gal or Htt171-18Q and β-Gal. Histological evaluation was carried out 12 weeks after infection using DARPP-32 immunohistochemistry. *A*, representative photomicrograph showing the loss of DARPP-32 in the striatum of a young rat. *B*, loss of DARPP-32 in the striatum of an old rat. Black arrowheads indicate the area with loss of staining. *C*, quantification of the volume of DARPP-32-depleted striatum. The loss measured after infection with lenti-Htt171-18Q corresponds to the mechanical damage caused by the injection needle. Note that lesions produced by Htt171-82Q are (+40%) larger in old rats. Results are expressed as mean+/−SEM. *p<0.05, Htt171-82Q vs. Htt171-18Q; #, p<0.01, young vs. old, ANOVA and Bonferroni's *post hoc* test.

Striatal degeneration induced by mutant Htt was assessed using DARPP-32 immunohistochemistry (Fig. 6). At 12 weeks post-infection, in lenti-Htt171-18Q-infected rats, the loss of striatal DARPP-32 expression was limited, and restricted to the vicinity of the needle track. This volume was small (<0.23 µm^3^), and showed no significant difference between age groups. In contrast, a clear loss of striatal DARPP-32 expression (1.5–2.0 mm^3^) was observed in Htt171-82Q-injected striata relative to lenti-Htt-18Q-injected striata in both age groups. Interestingly, the volume of the DARPP-32-depleted region in Htt171-82Q-injected striata was significantly greater (+59.6%, p<0.05) in older rats than in younger rats. This suggests that mutant Htt produces a more severe dysfunction and/or degeneration in older rats.

### Aging increases striatal neuron loss induced by mutant Htt

**Figure 7:**
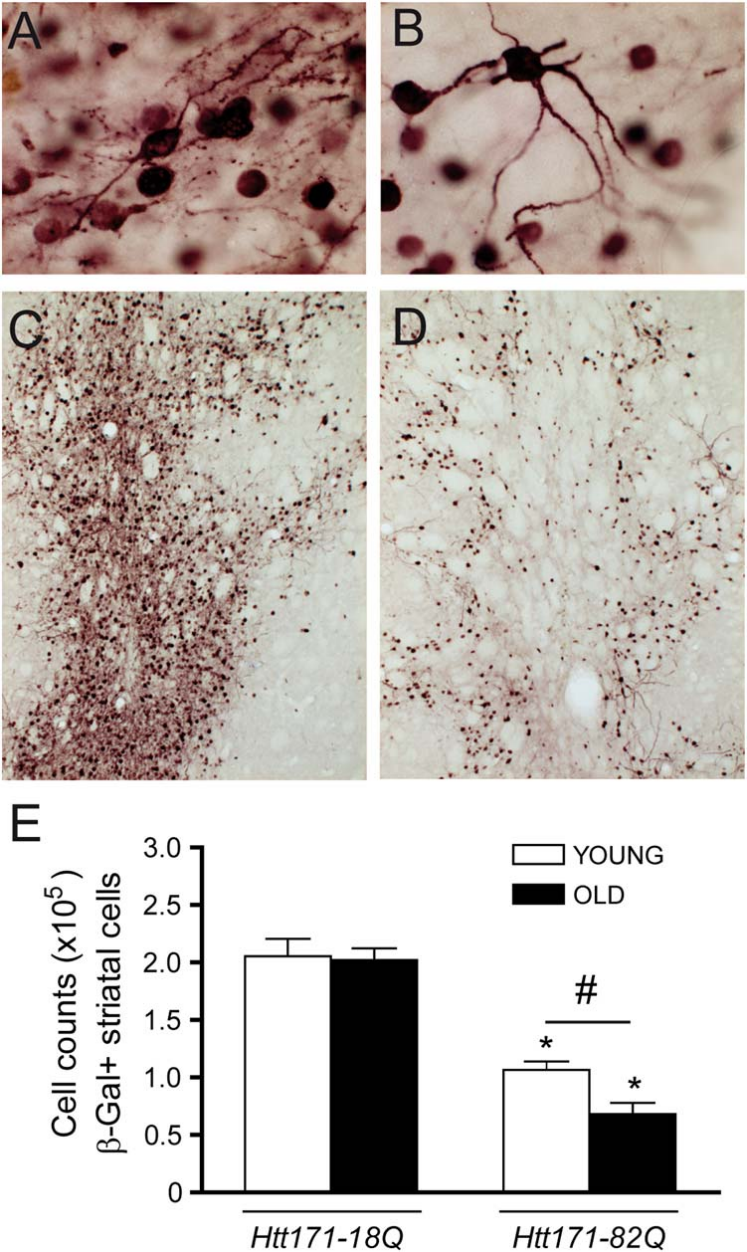
Htt171-82Q toxicity in exacerbated in old rats. Young (3 week) and old (15 month) rats received a stereotaxic injection of a mixture of lentiviral vectors (4 µl, 200 ng/µl of p24) encoding Htt171-18Q or Htt171-82Q and β-Gal. Immunohistochemistry for β-Gal was performed at 12 weeks post-infection. *A, B,* high magnification photomicrographs of β-Gal-positive neurons in a young rat co-injected with lenti-Htt171-18Q (*A*) and lenti-Htt171-82Q (*B*). Representative photomicrographs showing the repartition of β-Gal neurons in a young rat co-infected with lentiviral vectors encoding Htt171-18Q (*C*) or Htt171-82Q (*D*). Note the clear-cut loss of β-Gal-positive cells in the striatum co-injected with Htt171-82Q when compared with Htt171-18Q, consistent with the known toxicity of Htt171-82Q at this time point. *E*, stereological cell counts indicating the absence of age-dependent changes in the expression of β-Gal in cells co-infected with Htt171-18Q, whereas Htt171-82Q induces a greater loss of β-Gal+ cells in old rats than in young rats. Results are expressed as mean+/−SEM. *, p<0.05, Htt171-82Q vs. Htt171-18Q. #, p<0.01, young vs. old; ANOVA and Bonferroni's *post hoc* test.

We next assessed whether aging could actually modify striatal cell death induced by mutant Htt. For this purpose, we undertook unbiased stereological counts of neurons expressing β-Gal 12 weeks after intrastriatal co-injection of β-Gal vectors with vectors encoding Htt171-82Q or Htt171-18Q. By this method, most neurons (>90%) are transduced with both vectors, leading to the expression of both transgenes [20]. Cell counts of β-Gal+ cells in Htt-171-18Q-injected striata were not statistically different between young and old animals. In contrast, presumably as a result of neurodegenerative processes, the number of β-Gal+ cells in Htt-171-82Q-injected striata was significantly reduced (p<0.01) in both age groups as compared to Htt-171-18Q-injected striata (Fig. 7). The surviving β-Gal+ neurons were mainly restricted to the borders of the area infected by lenti-Htt-171-82Q, with the center of the infected area showing a markedly reduced density of β-Gal+ neurons. Importantly, the loss of β-Gal/Htt171-82Q–expressing striatal neurons in old rats was significantly greater (p<0.01) than that measured in young rats (Fig. 7).

## Discussion

Advanced age is generally associated with multiple cellular, structural and functional changes that may contribute to the age-associated decline of brain function [21]. Aging is generally considered a risk factor for neurodegenerative diseases, but its role in HD is unclear. Not only is this issue of fundamental interest, it also has implications for the development of therapeutic strategies. If aging processes exacerbate the alterations induced by mutant Htt, “normal” age-related cellular defects could constitute potential therapeutic targets for slowing HD progression.

In this study, we have obtained *in vivo* evidence that “normal” brain aging has an impact on the striatal toxicity of an N-terminal fragment of human mutant Htt. The reduction of DARPP-32 expression in the striatum of old rats, induced by a lentiviral vector encoding mutant Htt, indicates that these rats were more vulnerable to mutant Htt than young animals. In agreement with this, stereological cell counts demonstrate that the number of striatal neurons co-expressing β-Gal and mutant Htt is significantly reduced in aged rats. Interestingly, a quantitative analysis of brain sections from adult rats expressing mutant Htt shows that age affects the nature of the EM48+ aggregates. A detailed analysis of β-Gal expression indicates that the age-related modification of the toxicity of the N-terminal part of mutant Htt is not due to a difference in the efficiency of the PGK promoter between young and old rats. These observations suggest that the aged striatum is more vulnerable to the development of HD pathology.

The reduction of DARPP-32 immunoreactivity in our HD rat model probably results from a loss of expression of DARPP-32 in dysfunctional neurons and/or an actual loss of striatal neurons. DARPP-32, which is expressed by 96% of medium spiny striatal projection neurons, is down-regulated in the HD striatum [22], in HD transgenic mice [23], [24] and in a lentivirus-based HD rat model at 8 weeks post-infection [11]. DARPP-32 expression is regulated by the nuclear factor cAMP-response-element-binding protein (CREB). CREB signaling severely decreases with age in the striatum [25]. Many studies have reported that poly-Q expanded Htt interferes with transcriptional regulation involving CREB [26], [27]. It is thus possible that age-dependent alterations to CREB signaling may interact synergistically with CREB deficits induced by mutant Htt, increasing DARPP-32 loss and striatal degeneration in our rat model.

It is also possible that the apparent loss of β-Gal in striatal neurons expressing Htt171-82Q may at least in part result from alterations of transcription machinery. However, the effect of transcriptional deregulation might not be predominant. Indeed, the PGK promoter that drives β-Gal expression is also the promoter that drives mutant Htt expression in the same cells in our model. We observed strong accumulation of mutant Htt –containing nuclear inclusions in young and old rats, suggesting that mutant Htt is expressed at relatively high levels. Thus, if the loss of β-Gal phenotype was uniquely produced by transcription inhibition, mutant Htt expression would be markedly down-regulated as well, so that build up of inclusion/aggregates could not be detected. In previous studies, the loss of DARPP32 phenotype in our HD rat model has been correlated with loss of the neuronal marker NeuN, suggesting that it represents severe dysfunction and/or disappearance of striatal neurons and not only transcriptional alterations [11]. In line with this, neuroprotective strategies not directly targeting transcription (e.g. chaperones and calcineurin) block the loss of DARPP32 produced by mutant Htt in our rat model [12], [13], [61].

Thus the concomitant loss of DARPP32 and β-Gal in the present rat model is consistent with neurodegeneration (i.e. dysfunction and suffering) possibly without the actual disappearance of the striatal neurons. In addition, it may, at least in part, result from direct effects of transcriptional alterations caused by mutant Htt.

Our study also demonstrates that aging changes the characteristics of Htt-containing aggregates/inclusions. *In vivo*, lentiviral vectors mainly transduce neurons [28], [29]. The present results with lentiviral vectors encoding β-Gal support this view. In the HD brain, N-terminal fragments of Htt containing an expanded polyQ tract accumulate and form aggregates in the nucleus and neuropil of affected neurons [30]. In the R6/2 mouse, best studied model of HD, similar features have been observed [31]. The EM48 antibody is often used for the detection of aggregates/inclusions in *post mortem* tissue [16], [17]. With this antibody, the nuclei of neurons expressing mutant Htt show densely stained inclusions superimposed on diffuse staining (from light to dark staining depending on the cases) that encompasses the entire organelle. In contrast, somatodendritic/neuropil aggregates are generally small, and their background appears unstained [16], [19], [32]. In our experiments, both neuropil aggregates and intranuclear inclusions can be observed in striatal neurons expressing Htt171-82Q, as previously described in rats and non-human primates [11], [14]. Our results show that 4 weeks post-infection with lenti-Htt171-82Q, there is a clear-cut increase in the number of neuropil aggregates in older animals. There is also a subtle but significant reduction in the size of the nuclear inclusions. These observations suggest that age-dependent changes possibly affect the targeting/transport, elimination and/or sequestration of the N-terminal fragment of mutant Htt.

The relationship between the age-dependent changes in the aggregates/inclusions and the increase in vulnerability to mutant Htt is unclear. Nuclear polyQ inclusions are not correlated with neurodegeneration and may be a protective strategy developed by cells against mutant Htt toxicity [17], [33], [34]. In particular, Arrasate and collaborators [33] showed using video-microscopy analysis of striatal cells in culture that the accumulation of mutant Htt into large aggregates (“inclusion bodies”) correlates with improved survival. In old rats, we found that the larger polyQ aggregates (mostly nuclear inclusions) are slightly but significantly smaller than in the young rats. Thus it can be speculated that this change in old rats results from an inability to sequester toxic mutant huntingtin, leading to increased toxicity. In line with this, neuropil polyQ aggregates have been shown to be strongly correlated with disease progression [31], [32] and intimately associated with axonal degenerative processes [19], a hypothesis consistent with observations that Htt interacts with numerous proteins associated with cytoskeleton-based transport, such as HAP-1 [35]–[38]. The remarkable study by Wang and collaborators [39] further supports the view that neuropil aggregates are toxic to striatal neurons. In the present case, the increase in the number of small neuropil aggregates in old rats directly demonstrates the existence of an age-dependent modification(s) in mutant Htt processing. Whether this leads to increased toxicity in vivo still needs to be determined.

What defects might be involved in the age-dependent increase in mutant Htt toxicity? It is tempting to speculate that the pathways/components that are altered in both normal aging and mutant Htt toxicity could participate in the process. There seem to be several similarities between HD and aging. One major theory of aging is related to mitochondrial dysfunction and oxidative stress [5], [8], [40]. This is based on several types of observations including an age-dependent accumulation of mutations in mitochondrial DNA, [41], and the oxidative stress markers 8-hydroxy-2-deoxyguanosine (8OHdG) in both nuclear [42] and mitochondrial DNA [43], [44]. In addition, several genetic models have shown that alterations to the molecular machinery regulating the production or detoxification of reactive oxygen species (ROS) shortens life span [40], [45]. Oxidative stress has also been suggested to play a role in HD pathogenesis. In transgenic mouse models of HD (R6/2), there is an age-dependent increase in the levels of 8OHdG [46], [47], and high doses of the antioxidant ubiquinone (also known as CoQ10) increase the survival of HD transgenic mice [48], [49]. Thus, mitochondrial dysfunction and oxidative stress linked to “normal” aging may, along with mutant Htt-induced mitochondrial defects, synergistically modulate HD pathogenesis [9], [50].

Apart from mitochondrial changes, there are numerous age-dependent mechanisms that are also involved in HD pathogenesis. Although it is beyond the scope of this manuscript to review these processes in detail, we would like to provide a few examples to illustrate this point. Aging alters p53 function [51] and p53-related mechanisms are clearly involved in HD, possibly leading to mitochondrial anomalies [52]. Sirtuins, which are also involved in longevity/aging at least in part through FoxO regulation, can modify HD pathogenesis [53]. In line with this observation, Sirtuin SIRT1 also regulates PGC-1α, another important target involved in metabolism and longevity [54]. PGC-1α knock-down leads to striatal damage and precipitates neurodegeneration in HD transgenic mice [55], [56]. Aging also leads to a reduction in proteasome efficacy [6]. The role of proteasome impairment in HD is central, and age-related proteasome deficits would further facilitate the accumulation of toxic mutant Htt fragments as well as the accumulation of other proteins normally degraded by the proteasome [57].

Our results suggest that the aging process is instrumental in HD pathogenesis. Targeting age-related changes might thus slow disease progression. Characterizing the mechanisms underlying the effects of aging in HD could provide important information and lead to the identification of new therapeutic candidates. The lentiviral approach used in the present study could similarly be used to examine the effects of aging on the toxicity of mutant proteins involved in other neurodegenerative diseases (e.g. PS1/APP for Alzheimer's disease, and DJ-1, PINK-1, and LRRK2 for Parkinson's disease).

## Materials and Methods

### Animals

Young (3 week-old) and older (14–16 month-old) male Sprague-Dawley rats (Charles River) were used. For simplification, 14–16 month-old rats are called 15 month-old rats. The animals were housed in a temperature-controlled room maintained on a 12 hr light/dark cycle. Food and water were available ad libitum. Experiments were performed in accordance with the European Community Council directive 86/609/EEC for the care and use of laboratory animals.

### Lentiviral vector production

The construction of SIN-W-PGK (mouse phosphoglycerate kinase 1) vectors encoding Htt171-18Q and Htt171-82Q has been previously described [11], as has the nls-LacZ construct [58]. Viral particles were produced in human embryonic kidney 293T cells using a four-plasmid system [58], collected by ultracentrifugation and suspended in PBS with 1% bovine serum albumin (BSA). The particle content of the viral batches was determined by ELISA for the p24 antigen (Gentaur, France). Viral particles were used at a concentration of 200,000 ng of p24 per µl in 0.1 M Phosphate Buffer Saline (PBS) with 1% BSA for intrastriatal injections.

### Injection of lentiviral vectors

Male Sprague-Dawley rats were anesthetized with ketamine/xylazine (75 and 10 mg/kg respectively; i.p.) and placed in a stereotaxic frame. Bilateral stereotaxic injections into the striatum were made using a 30 gauge blunt-tip steel needle connected to a 10 µl Hamilton syringe (Hamilton, Reno, NV) via a 30 cm polypropylene catheter. Viral suspensions from the same batch were injected into each striatum at 0.25 µl/min by means of an automatic injector (Stoelting, Wood Dale, IL), using the following coordinates: 0.8 mm rostral to *bregma*, 3.5 mm lateral to midline and 4.0 mm ventral to the skull surface, with the tooth bar set at 3.3 mm. At the end of the injection, the needle was left in place for 5 min, before being slowly withdrawn. Animals were killed at 4 and 12 weeks after injection, and brains processed for immunohistochemistry.

### Experimental design

In a first set of experiments, we sought to establish the effect of normal aging on the formation of neuropil aggregates and intranuclear inclusions by infecting the striatum of young and older rats with lentiviral vectors encoding expanded Htt (Fig. 1). Animals (n = 6 per group) received a 2 µl injection of a LacZ lentiviral vector encoding β-Galactosidase or mutant Htt (Htt171-82Q) into the left and the right striatum respectively. Animals were killed 4 weeks post-infection. In a second set of experiments, we explored the effect of normal aging on Htt-induced striatal neuropathology, using two approaches. First, we compared the loss of the striatal protein dopamine- and cyclic AMP-regulated phosphoprotein with molecular weight 32 kDa (DARPP-32). Second, neuronal cell degeneration in young and older animals was examined using stereological cell counts (Fig. 1). In this case, animals (n = 10 per group) received a 4 µl injection of a LacZ lentiviral vector mixed (ratio 2∶1) with a vector encoding the wild-type N-terminal Htt fragment (LacZ+Htt171-18Q) or expanded Htt (LacZ+Htt171-82Q) into the left and the right striatum, respectively. Animals were killed 12 weeks post-infection.

### Brain tissue processing for histological evaluation

After anesthesia with a sodium pentobarbital overdose, animals were transcardially perfused with phosphate buffer containing 4% paraformaldehyde (PFA) and 1.5% picric acid. Brains were then post-fixed in 4% PFA for 24 h, and then cryoprotected by immersion in 15% and 30% sucrose for 48 h. Coronal sections (thickness 40 µm) were cut at −22°C using a sliding microtome (Cryocut 1800; Leica Microsystems, Nussloch, Germany). Free-floating sections encompassing the entire striatum were serially collected and stored in antifreeze cryoprotectant solution at −20°C until immunohistochemical processing.

### Immunohistochemistry, immunofluorescence and enzymatic detection of β-Gal activity

Immunohistochemistry for EM48 (mouse monoclonal antibody, gift of Pr X.-J. Li, Emory University, and Chemicon Intl. Inc., diluted 1∶2000), DARPP-32 (rabbit polyclonal antibody, Chemicon Intl. Inc., diluted 1∶5000) and β-Galactosidase (rabbit polyclonal antibody, diluted 1∶3000 for immunohistochemitry and 1∶800 for immunofluorescence) was performed as previously described [11], [59]. Immunoreactivity was revealed using the Vectastain ABC Elite System (Vector, Burlingham, CA). The sections were mounted, dehydrated by passing through ethanol and toluene, and coverslipped with Eukitt. For immunofluorescence of β-Gal, Alexa 488 goat anti-rabbit secondary antibodies were used (Molecular probes). The activity of β-Gal was revealed by incubating section for 2 h at 37°C in dionized water containing 4 mM potassium ferrocyanide, 4 mM potassium ferricyanide, 40 mM MgCl_2_, and 5-bromo-4-chloro-3-indolyl-β-O- galactopyranoside (0.4 mg/ml).

### Detection and quantification of EM48 aggregates

The EM48 antibody has been raised against the first 256 amino acids of human Htt with the polyglutamine tract deleted, and specifically stains both Htt-containing nuclear inclusions and somatodendritic/neuropil aggregates [16]–[19]. Nuclear staining generally shows a round weakly immunoreactive structure with the superimposition of strongly stained inclusions. Neuropil aggregates are generally seen as small densely stained objects often organized to resemble neuronal processes. Morphological, numerical and other features of EM48-positive inclusions were obtained by scanning 10–12 coronal sections spread over the anterio-posterior extent of the striatum (inter-section distance: 400 µm), using a 20× objective on a Zeiss Axioplan2 Imaging microscope motorized for x, y and z displacements, and an image acquisition and analysis system (Morphostar, IMSTAR, Paris) [12], [60], [61]. Section lighting was similar for all acquisitions and image light homogeneity was automatically corrected using blank images. For each section, 200–300 contiguous images (pixel size, 0.4 µm×0.4 µm) were acquired. All images were segmented using the same light threshold, mask smoothing and object size filters. Up to 1000–5000 EM48-positive objects (depending on the experimental group considered) were detected. With this set-up, objects with an apparent cross-sectional area of over 2 µm^2^ could be reliably detected. Objects were attributed to classes/bins with a step size of 2 µm^2^ in order to obtain a distribution histogram, as previously reported [12]. The number of objects was expressed as mean±SD for each bin. For each animal, the total number of aggregates/inclusions in the striatum was calculated by dividing the number of aggregates actually counted by the sampling frequency (1/10). The volume of the striatum containing EM48-positive objects was calculated by the Cavalieri method [62], using the formula: volume = *d.*(a_1_+a_2_+a_3_ …), where *d* is the distance between serial sections (400 µm), and a_1_, a_2_, a_3_ etc. stand for the area containing EM48-positive objects within individual serial sections throughout the striatum.

For very high resolution imaging, a similar X–Y scanning procedure was carried out using a 50× objective. This was combined with an analysis of stacks of images acquired along 5 µm of the Z axis. For each stack, all images were back-projected along the Z axis to yield one composite image, allowing very small objects (0.1 µm^2^ and above) to be segmented and identified. Since this analysis was highly time consuming, it was performed in only 3 representative striatal sections each of young and older rats infected with lentiviral vectors.

### Determination of striatal DARPP-32-depleted volume

The volume of the striatum exhibiting a depletion of DARPP-32 staining was estimated as follows. The unstained area of all serial striatal sections (400 µm apart) was manually delineated using a 4× objective and an AX70 microscope (Olympus, Munster, Germany) coupled with an image analysis system. The volume was then calculated according to the principle of Cavalieri (see above).

### Image analysis of β-Gal expression levels

Sections stained for β-Gal enzymatic activity and β-Gal immunofluorescence were scanned at 10× objective using a Zeiss Axioplan 2 Imaging microscope equipped with a motorized stage and an image acquisition and analysis system image (FluoUp and Mercator softwares, Explora Nova, La Rochelle, France). Care was taken to optimize image acquisition and avoid image saturation. Based on similar principles as described for automated detection of EM48 objects, segmentation of β-Gal-positive objects was performed and, for every object segmented, the mean levels of fluorescence (grey levels) or mean levels of β-Gal-enzymatic activity (optical density) was determined. Mean value for each animal was calculated based on the analysis of approximately 3000 β-Gal-positive objects per rat.

### Stereological counting of β-Galactosidase-positive neurons

The optical fractionator method [63], [64] was used to obtain an unbiased stereological estimate of the total number of β-Gal-positive cells within the striatum. Cells were counted using an AX70 Olympus microscope equipped with a digital color camera, an x–y motorized stage controller, a microcator to measure stage movements along the z-axis with a precision of 0.5 µm, and CAST-GRID stereology software (Olympus, Denmark). The striatum was delineated using a 4× objective, in accordance with a rat brain atlas [65]. Section thickness (from 13–16 µm) was measured at three locations for each section analyzed. Sampling was performed bilaterally within the delineated areas with a 100× oil-immersion objective. The area of the counting frame was 3521.2 µm^2^ and dissector height was 8 µm with a guard zone of 2 µm from the surface of the section. The sampling areas were separated by x–y steps of 296.7 µm×296.7 µm, generating counts of 150–300 sampled cells per animal. In the present study, the mean coefficient of error (CE) of the estimates was 0.08. The total number of β-Gal-positive cells within the entire striatum was calculated according to the following formula: N_tot_ = *ΣQ* ^−^. 1/ssf. 1/asf. 1/tsf where *ΣQ* ^−^ is the number of sampled cells, ssf is the section sampling fraction, asf is the area of the sampling fraction and tsf is the thickness of the sampling fraction.

All histological data (surface, volume measurements and cell counts) were performed by an investigator blind to the age of the animals.

### Statistical analysis

All data were expressed as means+/−SEM. In the first set of experiments, an unpaired Student's t-test was used for the comparison between the two age groups. In the second set of experiments, where results obtained from the left and right striata were considered to be independent (Htt-19Q versus Htt-82Q) and both age groups were compared, a one-way ANOVA with multiple comparisons using the *post hoc* Bonferroni method was carried out using commercially available software (StatView® software, SAS Institute Inc., USA). For all statistical tests performed, a probability level of 5% was considered significant.
